# Comparison between problematic use of social media and YouTube to insomnia among Iranian adolescents: A mediating role of psychological distress

**DOI:** 10.1177/20552076241261914

**Published:** 2024-09-24

**Authors:** Elahe Jafari, Po-Ching Huang, Fatemeh Zanjanchi, Marc N. Potenza, Chung-Ying Lin, Amir H Pakpour

**Affiliations:** 1 Social Determinants of Health Research Center, Research Institute for Prevention of Non-Communicable Diseases, 113106 Qazvin University of Medical Sciences, Qazvin, Iran; 2 School of Physical Therapy, Graduate Institute of Rehabilitation Science, College of Medicine, Chang Gung University, Taoyuan, Taiwan; 3 Department of Psychiatry, Yale School of Medicine, New Haven, CT, USA; 4 Connecticut Mental Health Center, New Haven, CT, USA; 5 Connecticut Council on Problem Gambling, Wethersfield, CT, USA; 6 Child Study Center, Yale School of Medicine, New Haven, CT, USA; 7 Department of Neuroscience, 5755 Yale University, New Haven, CT, USA; 8 Wu Tsai Institute, 5755 Yale University, New Haven, CT, USA; 9 Biostatistics Consulting Center, National Cheng Kung University Hospital, College of Medicine, 34912 National Cheng Kung University, Tainan, Taiwan; 10 Department of Public Health, College of Medicine, 34912 National Cheng Kung University, Tainan, Taiwan; 11 Department of Occupational Therapy, College of Medicine, 34912 National Cheng Kung University, Tainan, Taiwan; 12 Institute of Allied Health Sciences, College of Medicine, 34912 National Cheng Kung University, Tainan, Taiwan; 13 Department of Nursing, School of Health and Welfare, Jönköping University, Jönköping, Sweden

**Keywords:** Addictive behaviors, internet addiction, psychological distress, sleep, social media, YouTube

## Abstract

**Objective:**

Problematic use of the internet has been linked to emotional and sleep concerns, although relationships with specific types of internet use are less well understood. YouTube, as an online platform with video-watching features, may attract individuals to spend considerable time, for those experiencing problematic use be termed problematic use of social media (PUSM) or problematic use of YouTube (PUY). Therefore, the present study investigated relationships between PUSM/PUY, psychological distress, and insomnia among the Iranian adolescents.

**Methods:**

An online survey comprising Bergen Social Media Addiction Scale, YouTube Addiction Scale, Depression, Anxiety, Stress-21 Scale, and Insomnia Severity Index recruited 1352 participants.

**Results:**

Results of Hayes’ Process Macro showed significant correlations between the two types of problematic use and insomnia, with psychological distress as a mediator (unstandardized coefficient = 0.096 and 0.100).

**Conclusion:**

The findings implied the effect of psychological distress in mediating the relationships of PUSM and PUY to insomnia.

## Introduction

Among various type of addictive behaviors,^1–3^ social media addiction, or in a conservative term, problematic use of social media (PUSM), is a specific type of problematic use of internet. PUSM involves excessive, compulsive, and habitual use of social media to the extent that it interferes significantly with other important life activities such as academic performance or physical or mental health.^4^ In 2023, approximately 59.9% of the total population uses social media with an average of 4.7 new individuals joining daily,^5^ with some studies estimating 18% of the total population experiencing addictive use of social media.^6^ PUSM has been linked to food addiction,^7^ sedentary behavior,^7^ low-life satisfaction,^8^ insomnia,^4^ and psychological distress.^4,7^ Findings suggest potential positive association of PUSM on the psychological distress of individuals,^4,7^ which was connected to negative coping style that had negative impacts, such as sleep disturbance.^9^ The rapid increased in use,^5^ along with the detrimental correlates,^7–9^ have made PUSM a global issue warranting additional study.

Over time, social media has changed arguably from a tool for social interaction to online platforms for gathering information.^10^ The present study adopted a broad definition to define social media as online applications that allow for the exchange of user-generated content^11^ and digital information^12^ including (but not limit to) text, pictures or videos among individuals. YouTube, first launched in 2005, is a social media platform particularly for sharing videos.^13^ Compared to other social media, YouTube emphasizes content browsing and creation sharing.^14^ However, with limited interactive functionalities with video creators, YouTube provides more content viewing features, thus can be considered more as a video streaming application.^15^ Statistics reported that YouTube has more than 122 million individuals actively using the platform daily and approximately one billion total video watch hours per day.^13^ The *Uses and Gratifications theory* describing the interaction between social media and massive communication suggests that people tend to use social media to satisfy inner needs.^16,17^ Both viewing and creating behaviors on YouTube were indicated to generate the online gratification, which is associated with psychological satisfaction and may impact a minor group of population to develop problematic use of YouTube (PUY).^14,18^

Insomnia, a sleep disorder involving difficulties in initiating or maintaining sleep,^19^ affects more than half of the general population worldwide.^19^ Insomnia has the reported associations psychological status involving depression and anxiety,^20^ and can correlate to physical conditions including hypertension,^21^ cardiovascular disease,^22^ and obesity,^23^ which may worsen the general health of affected individuals. Addiction on social media^4^ have been linked to insomnia with the involvement of psychological distress^20,24^ and may particularly impact the relatively young populations (e.g. adolescents,^4^ university students,^20^ or young adults^25^) and cause longer term negative effects.^21^ Therefore, investigations into insomnia are warranted.

As a relatively new phenomenon, we know very little about PUY as well as its potential influence. Not only the relationships between PUY and insomnia have not been investigated yet, the comparison between PUSM and PUY remains unclear. The newly developed instrument, *YouTube Addiction Scale* (YAS), has been validated for its psychometric properties.^15^ Therefore, the present study aimed to (i) investigate the influences of PUY on psychological distress and insomnia and (ii) compare the underlying associations with PSMU, among Iran adolescents. Accordingly, the YAS was adopted to investigate the relationships between (i) PUY and insomnia and (ii) PUSM versus PUY and insomnia. In addition, considering the empirical evidence showing problematic use could lead to psychological distress and psychological distress could lead to negative outcome such as sleep problems,^4,7,9,20,24^ a model investigating the mediating role of psychological distress between two types of problematic use and insomnia was constructed. Specifically, the present study intends to explore a possible (iii) mediating effect of psychological distress for these two associations, if observed.

## Methods

### Participants and recruitment

This cross-sectional study assessed online social media use among Iran adolescents between September and November 2023. Eight high schools were randomly selected from a list of all high schools in Qazvin. Inclusion criteria were an age of 13 to 18 years and using YouTube. A link containing a consent form, parent's consent, and study descriptions was sent via SHAD Channels (an educational software and communication platform launched by the Iranian Ministry of Education) to eight schools in Qazvin to invite all the potential participants. Eligible students who read the study aims and descriptions and agreed to participate were asked to upload a signed consent form from one of their parents. The study was conducted in accordance with the Declaration of Helsinki. Adolescents who were willing to participate and one of their parents provided informed consent before study began. The study protocol was approved by the ethics committee of Qazvin University of Medical Sciences (IR.QUMS.REC.1401.097).

### Research hypotheses

It was hypothesized that (i) PUY would correlate with psychological distress and psychological distress with insomnia and (ii) psychological distress would mediate the relationship between insomnia and PUY. Similar relationships were hypothesized for PUSM. That is, (iii) PUSM would correlate with psychological distress and psychological distress with insomnia and (ii) psychological distress would mediate the relationship between insomnia and PUSM.

### Measures

*The Bergen Social Media Addiction Scale (BSMAS)*^26^ includes six items rated on a five-point Likert-like scale (1 = very rarely, 5 = very often) and was used to assess the level of PUSM. Scores were summed and higher scores reflect more severe PUSM. A sample item is, “*You spend a lot of time thinking about social media or planning how to use it.*” The psychometric properties (including construct validity, concurrent validity, test–retest reliability, and internal consistency) of the BSMAS have been found to be satisfactory in prior research,^26^ and its internal consistency in the present study was acceptable (Cronbach's α = 0.806).

*The YAS*^15^ includes six items rated on a 5-point Likert-Like scale (1 = never; 5 = very often) and was used to assess the severity of PUY. Scores were summed and higher scores reflect more severe PUY. A sample item is, “*Do you feel restless or worried if you can't access YouTube videos for any reason?*”. The psychometric properties (including construct validity, concurrent validity, and internal consistency) of the YAS have been found to be satisfactory in prior research,^15^ and its internal consistency in the present study was acceptable (Cronbach's α = 0.903).

*The Depression, Anxiety, Stress-21 Scale (DASS-21)*^27^ includes 21 items rated on a 4-point Likert-Like scale (0 = never; 3 = almost always) and was used to assess psychological distress of individuals among three dimensions (i.e. depression, anxiety and stress). The DASS-21 adopted an unique scoring method by summing the item scores and multiplying by 2.^27^ Items 3, 5, 10, 13, 16, 17, and 21 were used to calculate scores of the depression subscale; items 2, 4, 7, 9, 15, 19, and 20 were used to calculate scores of the anxiety subscale; and items 1, 6, 8, 11, 12, 14, and 18 were used to calculate scores of the stress subscale. The total scores ranged from 0 to 126, and higher scores reflect more severe psychological distress. A sample item is, “*I felt I was close to panic.*” The psychometric properties (including construct validity, concurrent validity, test–retest reliability, and internal consistency) of the DASS-21 have been found to be satisfactory in prior research,^27^ and its internal consistency in the present study was acceptable (Cronbach's α = 0.927).

*The Insomnia Severity Index* (ISI)^28^ includes seven items rated on a 5-point Likert-like scale (0 = none or very satisfied or not at all; 4 = very severe or very dissatisfied or very much) and was used to assess the severity of insomnia. The scores were summed, and higher scores reflect more severe insomnia. A sample item is, “*How SATISFIED/DISSATISFIED are you with your CURRENT sleep pattern?*” The psychometric properties (including construct validity, concurrent validity, test–retest reliability, and internal consistency) of the ISI have been found to be satisfactory in prior research,^28^ and its internal consistency in the present study was acceptable (Cronbach's α = 0.863).

### Data analysis

Descriptive statistics, including mean (SD) and frequency (%) were used to summarize participant characteristics and their scores on the measures. Confirmatory factor analysis was used to examine if all the measures used in the present study are sound. Specifically, the BSMAS, YAS, and ISI were fitted with a one-factor structure and the DASS-21 was fit with a three-factor structure. When fit indices are satisfactory (i.e. comparative fit index [CFI] > 0.9, Tucker-Lewis index [TLI] > 0.9, root-mean-square error of approximation [RMSEA] < 0.08, and standardized root mean square residual [SRMR] < 0.08), the four measures are considered to be sound for the following analyses. Pearson correlations were used to examine associations between the study variables (i.e. psychological distress, PUSM, PUY, and insomnia). Lastly, Hayes’ Process Macro^29^ was used to examine the mediating effects of psychological distress in the associations between the two types of problematic use (i.e. PUSM and PUY) and insomnia. The Model 4 in the Hayes’ Process Macro was used to test PUSM and PUY separately to avoid collinearity between the two types of problematic use. Specifically, two mediation models were constructed with the following settings. The first mediation model had PUY (i.e. the YAS score) as a predictor, psychological distress (i.e. the DASS-21 score) as a mediator, and insomnia (i.e. ISI score) as an outcome with age and gender controlled. The second mediation model had PUSM (i.e. the BSMAS score) as a predictor with the same mediator, outcome, and controlled variables as in the first mediation model. For assessing the mediating effect (also known as indirect effect), a bootstrapping method was used to determine statistical significance: when the 95% bootstrapping confidence interval of a mediating effect does not cover 0 among the 5000 bootstrapping resamples, the mediating effect is considered significant.

## Results

Among the 1352 participants (mean [SD] age 15.7 [1.4] years; 46.1% males) who completed the survey, they had a mean score of 12.03 (SD = 5.65) on the YAS, 16.48 (SD = 7.82) on the BSMAS, 31.86 (SD = 16.40) on the DASS-21, and 10.74 (SD = 6.23) on the ISI (Table 1). The YAS had satisfactory fit in its one-factor structure (χ^2^ [df] = 40.016 [9], CFI = 0.986, TLI = 0.975, RMSEA = 0.080, and SRMR = 0.017); the BSMAS had satisfactory fit in its one-factor structure (χ^2^ [df] = 28.833 [9], CFI = 0.996, TLI = 0.994, RMSEA = 0.067, and SRMR = 0.039); the DASS-21 had satisfactory fit in its three-factor structure (χ^2^ [df] = 440.077 [186], CFI = 0.992, TLI = 0.991, RMSEA = 0.052, and SRMR = 0.058); and the ISI had satisfactory fit in its one-factor structure (χ^2^ [df] = 43.010 [14], CFI = 0.985, TLI = 0.978, RMSEA = 0.064, and SRMR = 0.063). The satisfactory fit for all the measures supports the following analyses. Associations between all study variables (i.e. psychological distress, PUSM, PUY, and insomnia) had Pearson correlations ranging between 0.249 and 0.463 (all *p*-values < 0.001; Table 2).

**Table 1.** Characteristics of the studied participants (N = 1352).

	Mean (SD) or n (%)
Age (year)	15.74 (1.40)
Gender	
*Male*	623 (46.1%)
*Female*	729 (53.9%)
Bergen Social Media Addiction Scale (BSMAS) score	16.48 (7.82)
YouTube Addiction Scale (YAS) score	12.03 (5.65)
Depression, Anxiety, Stress-21 (DASS-21) score	31.86 (16.40)
DASS-21 Stress subscale score	12.63 (6.70)
DASS-21 Anxiety subscale score	10.74 (6.23)
DASS-21 Depression subscale score	8.02 (5.69)
Insomnia Severity Index (ISI) score	10.74 (6.23)

**Table 2.** Pearson correlation matrix of the studied variables.

	***r***			
	**1.**	**2.**	**3.**	**4.**
1. Psychological distress^**a**^	–	0.398	0.249	0.463
2. Problematic use of social media^**b**^		–	0.401	0.417
3. Problematic use of YouTube^**c**^			–	0.368
4. Insomnia^**d**^				–

^a^Assessed using Depression, Anxiety and Stress-21 Scale (DASS-21).

^b^Assessed using Bergen Social Media Addiction Scale (BSMAS).

^c^Assessed using the YouTube Addiction scale (YAS).

^d^Assessed using a Insomnia Severity Index (ISI).

Note: All *p*-values < 0.01.

**Figure 1.**
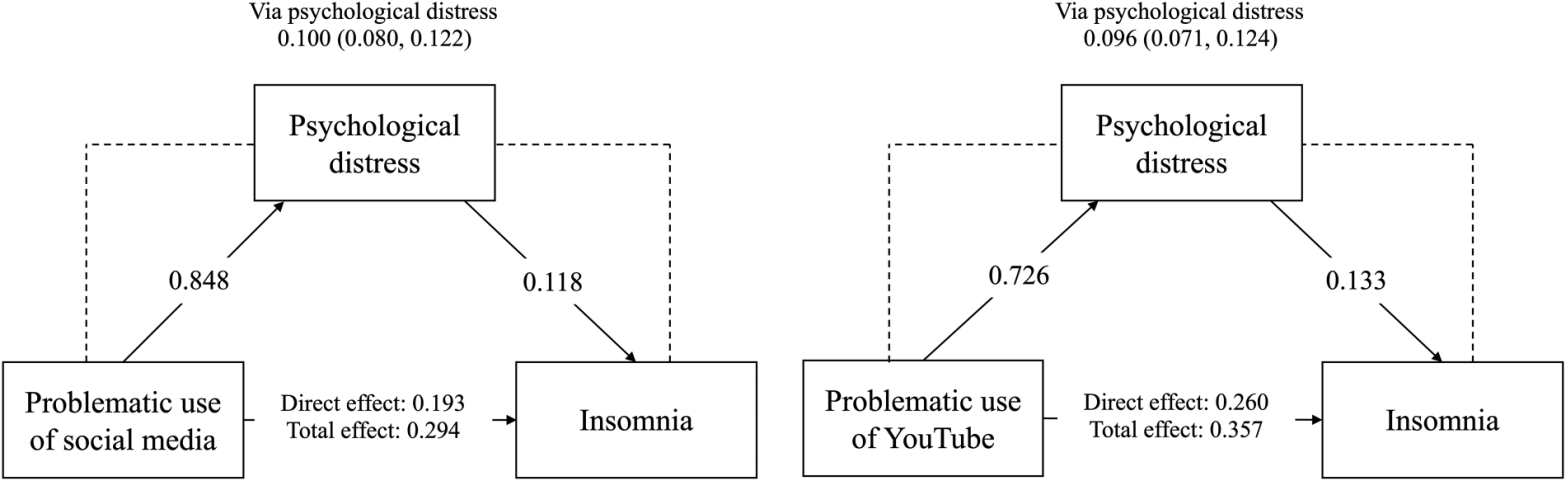
Results of proposed mediation models. *Notes*. Age and gender were controlled. Solid lines indicate direct effects and data was presented as unstandardized coefficient with all *p* < 0.001. Dashed lines indicate indirect effects via the mediator (i.e. psychological distress) and data was presented as unstandardized coefficient with 95% bootstrapping confidence interval.

In mediation analyses, both PUSM and PUY had significant direct effects on insomnia (unstandardized coefficient [B] = 0.193 for PUSM and 0.260 for PUY, all *p* < 0.001) and psychological distress (B = 0.848 for PUSM and 0.726 for PUY, all *p* < 0.001). In addition, psychological distress mediated associations between the two types of problematic use (PUSM and PUY) and insomnia (Table 3). Specifically, the unstandardized indirect effect of PUY on insomnia through psychological distress was 0.096 (95% bootstrapping confidence interval: 0.071, 0.124). The unstandardized indirect effect of PUSM on insomnia through psychological distress was 0.100 (95% bootstrapping confidence interval: 0.080, 0.122). The results of the proposed mediation models are shown in Figure 1.

**Table 3.** Models that tested mediated effects of psychological distress on insomnia.

	Unstand. Coeff.	SE or (Bootstrapping SE)	t-value or (Bootstrapping LLCI)	*p*-value or (Bootstrapping ULCI)
PUY as a predictor				
total effect of PUY on insomnia	0.357	0.025	14.073	<0.001
Direct effect of PUY on insomnia	0.260	0.024	10.909	<0.001
Direct effect of PUY on mediator (i.e. psychological distress)	0.726	0.079	9.237	<0.001
Indirect effect of PUY on insomnia through psychological distress	0.096	(0.013)	(0.071)	(0.124)
PUSM as a predictor				
total effect of PUSM on insomnia	0.294	0.018	16.392	<0.001
Direct effect of PUSM on insomnia	0.193	0.018	10.575	<0.001
Direct effect of PUSM on mediator (i.e. psychological distress)	0.848	0.054	15.740	<0.001
Indirect effect of PUSM on insomnia through psychological distress	0.100	(0.010)	(0.080)	(0.122)

Note: Age and gender were adjusted for the model.

PUY = problematic use of YouTube; PUSM = problematic use of social media; Unstand. Coeff. = unstandardized coefficient; SE = standard error; LLCI = lower limit in 95% confidence interval; ULCI = upper limit in 95% confidence interval.

## Discussion

The present study investigated relationships between two types of problematic use (i.e. PUSM and PUY) and insomnia, as well as mediating effects of psychological distress, among the Iranian adolescents. Both types of problematic use (i.e. PUSM and PUY) significantly correlated with insomnia, with psychological distress mediating the relationships. Thus, proposed hypotheses were supported. Implications are discussed below.

Correlations between general social media use and insomnia have been previously reported.^4,30^ The present study extended this association to the specific PUY. One study reported that people tended to use YouTube for seeking information and knowledge, but not passing time or entertainment purposes,^18^ which matches with the theory of Uses and Gratification that YouTube provides a sense of online satisfaction.^14^ Further, binge viewing was reported to correlate with insomnia as cognitive pre-sleep arousal being an explanatory factor. The findings suggest that a need for information-seeking may motivate the use of social media or YouTube to review online content, subsequently increasing the arousal levels, negatively impacting overall sleep quality, and leading to insomnia. Moreover, the influence of artificial light in affecting circadian rhythms and generating insomnia should be considered.^31^

This study corroborated relationships between psychological distress and PUSM,^4,7^ and further verified its existence in PUY. Individuals with social anxiety and loneliness may use social media to compensate for insufficient social relationships^32^ because social interactions on the internet not only provide interpersonal satisfaction,^14^ but may be less stressful.^33^ However, passively use of social media (i.e. only scrolling and browsing the content without any interaction) may provide a platform for social comparison and jealousy,^34^ which may worsen psychological well-being.^35^ Moreover, a study showed that parasocial relationships (i.e. an one-side relationships formed by one party and not known by the other) mediated the association between social anxiety and PUY, and social anxiety moderated the correlation between parasocial relationship and PUY,^33^ suggesting that parasocial relationships may worsen social anxiety and further promote the development of PUY. Accordingly, the perceived social anxiety may increase the time spent on YouTube, which strengthens associations between parasocial relationships and PUY. These factors should be examined further in future studies.

Psychological distress mediated associations between two types of problematic use and insomnia. Both psychological distress and insomnia correlated with PUSM,^4,7,36–41^ although the directionalities and interaction among these three factors needed further investigation. Several studies have suggested the mediation effect of sleep quality in the associations between social media use and psychological health.^37–39^ However, we also found other studies supported the mediation effect of psychological health in the correlation between insomnia and addictive use of social media,^40,41^ which was supported by the present finding. Individuals with social disadvantage were frequently reported to associate with psychological distress^35^ and may use social media to escape from reality.^17,42^ An quantitative study clearly reported that online video watching may provide virtual social interaction and a sense of reassurance, but individuals may also gain the feel of boredom and hollowness after watching, which formed a vicious cycle in exacerbating the psychological distress.^42^ As a result, the biological change derived from psychological distress (e.g. elevated cortisol level) may disrupt the sleep quality^41^ and worsen the insomnia.

The difference between YouTube and traditional social media (e.g. Facebook) is that YouTube focuses on video content,^14,15^ which endorses a participatory culture and allows individuals to create their own community and share their opinion or experience.^43,44^ However, the YouTube recommender system may also lead to problematic content, such as such as violent extremism or misleading information, which may be detrimental.^45^ With rapidly increased use of YouTube, PUY warrants more investigation and practical solutions. From the present finding, for those with PUSM or PUY, close monitoring of psychological states and sleep quality, as well as providing self-regulation techniques and instructions on sleep hygiene,^4^ may help reduce negative influences. For those with particular PUY, communication and interaction skills can be taught to those with poor social relationships in order to facilitate real-life social interactions^32^ and fulfill the psychological and interpersonal satisfaction.^14^

The present study has several limitations. First, the use of self-reported questionnaires may result in social desirability bias (e.g. participants may report less time using social media and YouTube) or recall bias (e.g. participants may underestimate time spent on social media). Second, the cross-sectional study design cannot provide insight into the cause-and-effect relationships between variables. Third, the role of participants on YouTube (whether they were viewers or creators) had not been investigated. Therefore, we may not provide the information regarding the participants’ activity on YouTube. Fourth, the habits of media use, such as time spent on media or the purposes of using the media, were not investigated. Therefore, the mechanism of media use in affecting sleep quality remains unclear and warrants further investigation. The effect of the corona virus 2019 (COVID-19) pandemic cannot be excluded. As a worldwide pandemic, COVID-19 was reported to impact the study variables (i.e. social media use, psychological distress, and insomnia).^46^ Accordingly, the potential influence of the COVID-19 pandemic should be considered.

Future studies may either (i) focus on the impacts of users’ patterns, such as time spent on media or participate types (active participants such as creators or passive participants such as viewers), on negative outcomes (e.g. insomnia or addictive behaviors), or (ii) investigate the risk and protective factors in developing PUY, as well as the possible YouTube addiction. The information may further be implemented in developing strategies to protect the individuals from negative consequences of excessive media use.

## Conclusion

The present study investigated correlations between two forms of problematic use (i.e. PUSM and PUY) and insomnia among the Iranian adolescents. Additionally, a mediation effect of psychological distress was examined. The results showed that both PUSM and PUY were correlated with insomnia, and psychological distress mediated the relationships. Accordingly, strategies such as monitoring psychological status and sleep quality, as well as self-regulation or sleep hygiene techniques, may be adopted by and directly examined in people with problematic use. Particularly, practical communication skills may be taught to those potential PUY with poor social relationships to facilitate real-world social interactions.
